# Can kefir supplementation influence the growth performance and serum biochemistry of artificially reared squabs of three pigeon breeds?

**DOI:** 10.5194/aab-69-529-2026

**Published:** 2026-09-30

**Authors:** Hande Işıl Akbağ, Atakan Şensoy, Hakan Erdem, Soner Yiğit, Cemil Tölü, Türker Savaş

**Affiliations:** 1 Department of Animal Science, Faculty of Agriculture, Çanakkale Onsekiz Mart University, Çanakkale, Türkiye

## Abstract

This study aimed to evaluate the effects of kefir on growth performance and some serum parameters in artificially reared squabs. A total of 114 1 d old squabs (Takla, Dolapçı, and Homing breeds) were fed crop milk by their parents for 3 d. The squabs were then randomly assigned to one of three treatment groups: (1) the control group, which was fed naturally by their parents; (2) the artificial crop milk (ACM) group, which was hand-fed with artificial crop milk; and (3) the kefir (KEF) group, which was hand-fed with artificial crop milk supplemented with 2 % kefir. Samples of crop milk were collected 3 d after hatching, and their chemical composition was analysed. The body weights and feed intake of the squabs were monitored daily. Blood samples were taken from the squabs at 28 d of age to determine serum biochemical parameters. The nutritional composition of crop milk did not vary according to pigeon breed ( P>0.05 ). In the study, it was determined that body weight, body weight gain, feed intake, and feed conversion ratio were significantly reduced in the ACM and KEF groups compared to the CON group ( P≤ 0.05). In the KEF group, Homing pigeon squabs had higher feed intake and body weight than those in the ACM group. Serum biochemical parameters, except for aspartate aminotransferase, differed among the pigeon breeds ( P≤ 0.05). In contrast, among the measured serum parameters, only total protein and albumin were significantly affected by the dietary treatments ( P≤ 0.05). In conclusion, hand-feeding with artificial crop milk resulted in poorer growth performance than parental feeding.

## 1 Introduction

Historically, pigeons have been kept for a variety of reasons, including their emotional, religious, and cultural significance. However, their primary function was to serve as messengers (Kokoszyński et al., 2020). Pigeons were also kept for their meat in many homes (Maity et al., 2020). The first meat pigeon was developed in the United States during the 1980s, after which the world meat pigeon industry began to grow gradually (Kabir, 2021).

Pigeons are altricial birds, and their only source of nutrition in the first days of life is the crop milk produced by their parents (Dumond, 1965; Sales and Lanssens, 2003). Pigeon squabs grow well thanks to the rich source of lipids, proteins, and other bioactive factors found in crop milk (Zhu et al., 2022). In the first week of life, squabs primarily depend on pigeon crop milk, with the proportion of feed provided by their parents increasing from 4 to 12 d of age (Vanderputte-Poma, 1980). Although significant research has examined the mechanisms involved in producing “milk” from pigeon crops, the main components of crop milk remain unclear. Furthermore, the unique feeding habits of pigeon squabs hinder the pigeon industry's intensive breeding capacity (Ding et al., 2020).

In terms of growth performance and immunity, early-weaned squabs were less healthy than those fed by their parents (Wen et al., 2022). Squabs that are fed artificial crop milk have been reported to have very low body weights, and deaths have even been observed (Omar et al., 2014). This suggests the presence of some growth factors necessary for the development of squabs, in addition to the high nutritional content of crop milk. It has been suggested that the observed decline in mortality and growth rates is due to the presence of bioactive proteins and growth factors, as well as abundant microbiota, including *Lactobacillus*, *Enterococcus*, *Veillonella*, and *Bifidobacterium*, in crop milk (Ding et al., 2020). Furthermore, it has been reported that the microbiota obtained from crop milk during the initial post-hatch phase has a positive impact on the development of humoral immunity and growth in the squabs (Jacouin et al., 2012).

Kefir is a fermented beverage belonging to the probiotic class. It contains lactobacilli, streptococci, and yeasts (Otles and Cagindi, 2003), as well as proteins, polysaccharides, ethanol, lactic acid, fat, vitamins, and minerals (Magalhaes et al., 2011). It has been reported that the oral administration of kefir has a beneficial effect on the growth performance of broiler chicks. (Cho et al., 2013). Various bioactive compounds, such as kefiran, are formed during the fermentation of milk with kefir grains. These compounds are similar to peptides and heteropolysaccharides and have also been reported to exhibit antioxidant and antimicrobial properties (Kim et al., 2019). Considering the microbiota, bioactive peptides, and heteropolysaccharides it contains, pigeon's crop milk has similarities with kefir in terms of structure and effects on the organism (immune strengthening and growth stimulating).

The majority of studies on crop milk in the literature aim to evaluate its chemical and microbiological composition and the effects of crop milk on immunity. We are unaware of any studies that have evaluated the effects of feeding pigeon squabs artificial crop milk and using kefir. The study hypothesizes that squabs consuming kefir in conjunction with artificial crop milk will demonstrate comparable growth performance to squabs raised by their parents.

The purpose of this study is to determine how kefir and artificial crop milk together affect the growth performance and certain blood parameters of pigeon squabs.

## 2 Material and methods

### 2.1 Animals

A total of 60 pigeons, including 10 pairs of the Homing pigeon, 10 pairs of the Talka breed, and 10 pairs of the Dolapçı breed, were used in the study. Homing pigeons, once used for message delivery, were even the first “drones” used for aerial photography (O'Hagan and Serafinelli, 2022). Today, these pigeons are used in racing competitions, where they fly relatively long distances. The Dolapçı pigeons and the Talka pigeons, also known as Takla, are native breeds of Turkey that are popular because of their flight behaviour – tumbling and spinning (Yılmaz et al., 2013). Pigeons were obtained from three local pigeon fanciers. The squabs included in the experiment were divided into three groups: the first group was fed by their parents (control group, CON, n= 31), the second group was hand-fed with artificial crop milk (artificial crop milk group, ACM, n= 42), and the third group was hand-fed with artificial crop milk added with kefir (kefir group, KEF, n= 41). The number of squabs allocated to each breed within each dietary treatment was as follows: in the control group, there were 11 Dolapçı, 11 Mardin, and 9 Posta squabs; in the kefir group, there were 20 Dolapçı, 11 Mardin, and 10 Posta squabs; and in the artificial crop milk (ACM) group, there were 18 Dolapçı, 12 Mardin, and 12 Posta squabs.

### 2.2 Feeds used in feeding breeder pairs and squabs

During this experiment, breeder pairs were fed with a 55 % commercial pelleted layer chicken feed (16.9 % CP and 3200 kcal g^−1^) and 45 % wheat grain (12.8 % CP and 3000 kcal g^−1^) mixture ad libitum. Röhnfried's artificial crop milk (Germany) and Altinkılıç kefir (Çanakkale, Türkiye) were utilized in the nutrition of the hand-fed squabs. The chemical composition of the experimental feeds is shown in Table 1.

**Table 1:** Chemical composition of pellet feed and wheat grain mixture used in parents' nutrition.

Item	DM %
Dry matter	90.95
Crude protein	14.69
Neutral detergent fibre	23.21
Acid detergent fibre	9.46
Acid detergent lignin	1.90
Ash	5.78

Table 2 shows the chemical composition of artificial crop milk and kefir used to feed hand-fed squabs in the study. The Altınkılıç company has reported the microbiological composition of kefir as *Lactococcus* spp. (10^8^ cfu g^−1^), *Lactobacillus* spp. (4.7 × 10^8^ cfu g^−1^), and yeast (10^3^ cfu g^−1^).

**Table 2:** Chemical composition of artificial crop milk and kefir used in hand-feeding of squabs, DM %.

Item	Artificial	Kefir
	crop milk	
Dry matter	89.59	12.00
Crude protein	26.02	3.83
Ether extract	6.70	2.42
Crude cellulose	2.79	0.00
Ash	9.25	1.20

### 2.3 Care of breeder pairs and squabs

Male and female pigeons were kept separately to get used to the trial room and the cages in which they would be placed during the experiment. After 1 week, the pigeons were put in the same cage with their mates. In the study, parents and squabs cared for by their parents were housed in cages (50 × 45 × 45 cm) containing a plastic feeder, a plastic waterer, and a nest. To reduce the difference in hatching time between the two eggs, after the first egg was laid, the first egg was taken, and a dummy egg was left in its place, and after the second egg was laid, the first egg was placed back in the nest. The animal material of the experiment was obtained by consecutive hatching (four in the Dolapçı pigeons and five in the Homing pigeons and Takla). The control group squabs were fed by their parents until 28 d of age. Squabs from the group ACM and KEF stayed with their parents for 3 d after hatching. At the end of the third day (at 16:00 UTC +3 ), they were taken to the chick-rearing box. The environmental temperature between 3 and 15 d old was 38 °C, and between 16 and 28 d old it was 25 °C. The squabs in the ACM group were fed with a mixture of artificial crop milk and water in a 1 : 1 ratio three times a day (09:00, 12:00, and 16:00) from 4 d of age to 10 d of age. The temperature of the artificial crop milk was adjusted to body temperature (39 °C). The mixture was administered directly in the crop of the squabs using a crop catheter until their crops were full. From 10 to 12 d of age, the squabs were fed an equal amount of artificial crop milk, as well as a mixture of the feed consumed by the parents, which had been ground and diluted with water at a ratio of 1 : 2. From 12 to 28 d old, the squabs were fed with a feed mixture prepared for their parents and mixed with water in a 1 : 2 ratio. They were fed twice a day at 09:00 and 16:00. Kefir was included in the feed of the squabs in the KEF group at a rate of 2 % (at a level 2 % of the daily feed intake) from 4 to 28 d of age. The hand-fed squabs were given the same amount of feed in the afternoon as they consumed in the morning. Breeder pairs were fed once a day at 09:00 in the morning. The feed intake of the squabs was calculated as the difference between the weight of the squabs before and after feeding. Feed intake of squabs was measured. To determine the feed conversion ratio (FCR) of the squabs, the total feed intake (g) was divided by the total weight gain (g). The mortality rate is the proportion of deaths among squabs between 3 and 28 d old.

### 2.4 Chemical analysis of feed and crop milk samples

Samples of the crop milk were collected 3 d after the squabs hatched. A plastic catheter was used to extract milk samples from the squabs' crop 3 h after the parents had been fed. Samples were placed into 15 mL falcon tubes and stored at - 20 °C until analysis day. Dry matter (DM), ether extract (EE), crude protein (CP), and ash analyses were performed on crop milk and feed samples according to the methods reported by AOAC (2000). The ANKOM 200 fibre analyser was used to analyse feed samples following the Van Soest et al. (1991) method for neutral detergent fibre (NDF), acid detergent fibre (ADF), and acid detergent lignin (ADL) analysis. The method reported by Dubois et al. (1956) was used to analyse the water-soluble carbohydrate (WSC) concentration in crop milk.

### 2.5 Blood sampling and analysis

The veins under the wings were tapped for blood sampling when the squabs were 28 d old. The serum of the blood samples was separated for 10 min by centrifuging at 3500 rpm at 15 °C; then they were kept in a deep freezer at - 20 °C until the day of analysis. Serum samples were analysed for total protein (TP), albumin (ALB), total cholesterol (CHOL), glucose (GLU), alanine aminotransferase (ALT), and aspartate aminotransferase (AST) using commercial kits (Improgen Diagnostic Chemical Company, İstanbul, Türkiye) with a biochemical analyzer (Urit-880, Jiangsu, China).

### 2.6 Microbiological composition of crop milk

Crop milk samples were incubated at 30 °C for 3 d using TGYE (tryptone glucose yeast extract agar) to develop total aerobic bacteria, as indicated by TS EN ISO 4833-1 (TSE, 2014). Petri dishes were placed at 25 °C for 5 d, and yeasts were developed using DRBC (dichloran rose bengal chloramphenicol) agar (TS EN ISO 21527-1, TSE, 2012). To ensure the growth of lactic acid bacteria (LAB), crop milk samples were incubated at 30 °C for 3 d on MRS (de Man, Rogosa and Sharpe) agar in accordance with the standard (TSE, 2004).

### 2.7 Statistical analysis

In the analysis of body weight, feed intake, feed conversion rate, and blood serum analyses, the method of repeated measurement variance analysis was employed with a statistical model involving group, breed, observation day, and interactions. In the analysis of crop milk chemical and microbiological analysis, the variance analysis method was employed, and the model included group, breed, and interaction. The quantities of yeast, lactic acid bacteria, and aerobic bacteria counts were converted to the coliform unit (cfu g^−1^) by logarithmic transformation. In the statistical analysis, the variance analysis method was employed, which included breed, group, and their interaction as fixed factors. The Tukey test was employed in post hoc analyses. The SAS (2014) package programme was utilized in all analyses. The mean values presented in the tables are the least squares means with their standard errors

## 3 Results

### 3.1 Quality parameters of crop milk

The chemical composition of crop milk samples according to breed is shown in Table 3. The chemical composition of crop milk samples was not affected by the breed ( P>0.05 ). It has been observed that breeds have similar average values in terms of the chemical composition of crop milk.

**Table 3:** The chemical composition of pigeon crop milk obtained from different breeds in the study.

Breed	Dolapçı		Takla		Homing pigeons		P
Item	$\bar{x}$	SE	$\bar{x}$	SE	$\bar{x}$	SE	
Dry matter	19.24	0.88	18.71	1.22	17.01	1.49	0.4461
Crude protein	43.28	1.23	43.29	1.70	42.20	2.08	0.8949
Ether extract	29.68	1.31	27.46	1.80	32.02	2.20	0.2860
Crude ash	1.51	0.21	2.11	0.28	2.29	0.35	0.0914
Water-soluble carbohydrate	29.73	0.79	30.02	1.08	30.28	1.32	0.9332
Lactic acid	161.46	11.17	185.04	15.35	203.51	18.79	0.1476

Dry matter: %; crude protein: % DM; ether extract: % DM; crude ash: % DM; water-soluble carbohydrate: % DM; lactic acid: g kg^−1^ DM.

The total count of aerobic bacteria (TAB) and yeast in crop milk remained unchanged ( P>0.05 ), but the content of lactic acid bacteria (LAB) was different ( P=0.0481 ) depending on the breed. Among the breeds, the Homing pigeons had the highest average LAB (3.96 ± 0.19 ${\mathrm{log}}_{10}$ cfu g^−1^). The crop milk LAB content of Dolapçı (3.50 ± 0.19 ${\mathrm{log}}_{10}$ cfu g^−1^) and Takla (3.24 ± 0.19 ${\mathrm{log}}_{10}$ cfu g^−1^) pigeons was found to be similar, as can be seen in Table 4.

**Table 4:** Changes in the microbiological composition of crop milk according to pigeon breed.

Breed	Dolapçı		Takla		Homing pigeons		P
Item	$\bar{x}$	SE	$\bar{x}$	SE	$\bar{x}$	SE	
TAB	4.46	0.12	4.60	0.12	4.45	0.12	0.6076
LAB	3.50^b^	0.16	3.24^b^	0.17	3.96^a^	0.19	0.0481
Yeast	3.38	0.23	3.08	0.22	4.46	0.25	0.4848

Different letters within rows indicate statistically significant differences among breeds ( P≤ 0.05). TAB: total aerobic bacteria, ${\mathrm{log}}_{10}$ cfu g^−1^; LAB: lactic acid bacteria, ${\mathrm{log}}_{10}$ cfu g^−1^.

### 3.2 Performance and mortality of the squabs

It was determined that the body weights of squabs varied depending on the group, breed, observation day, and their interactions ( P<0.0001 ). Except for breed × observation day ( P=0.0635 ), other interactions on feed intake and FCR were found to be significant ( P<0.05 ). Figure 1 shows the changes in the weights of squabs according to the treatment group (Fig. 1a) and breeds (Fig. 1b).

**Figure 1:**
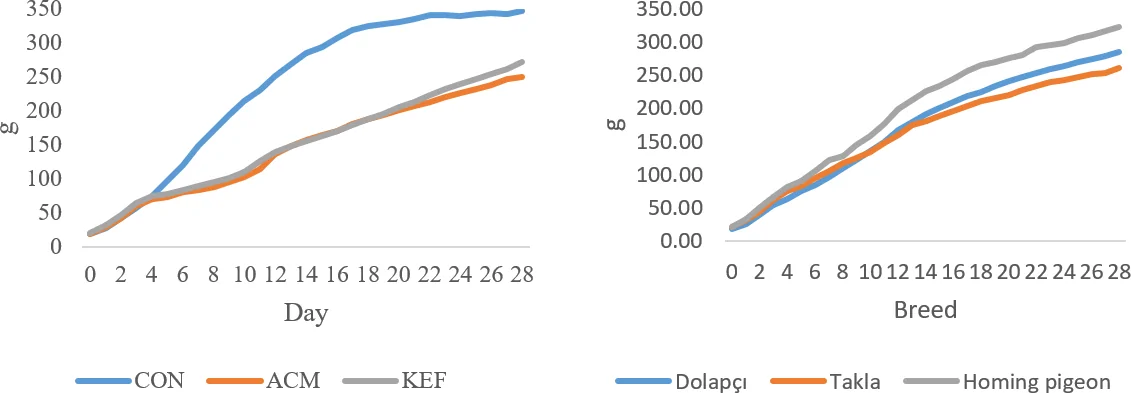
Changes in squabs' body weight by age, group **(a)**, and breed **(b)**.

The CON group (236.05 ± 1.074 g) has the highest overall weight in the groups, followed by the KEF (152.18 ± 1.039 g) and ACM groups (146.02 ± 1.041 g), respectively ( P≤ 0.05). In the study, the Homing pigeons had the highest overall body weight (199.29 ± 1.151), followed by the Dolapçı (171.40 ± 0.933) and Takla pigeons (163.55 ± 1.059), respectively.

Table 5 presents the body weights measured at 28 d of age (final) according to feeding treatments and breeds.

**Table 5:** Final LS (least squares) means of body weights (g) of squabs according to breed and treatments.

Breed	Group	$\bar{x}$	SE
Dolapçı	CON	304.45^b^	9.665
	ACM	279.00^ce^	8.567
	KEF	272.07^c^	8.277
Takla	CON	314.41^b^	9.665
	ACM	232.70^d^	10.137
	KEF	238.15^d^	10.137
Homing pigeon	CON	424.06^a^	10.685
	ACM	241.13^d^	11.333
	KEF	305.63^be^	11.333
P	< 0.0001		

The columns with different letters show a significant difference between breed x treatment subgroups ( P≤ 0.05).

Body weights at 28 d of age varied significantly according to breeds and treatments ( P<0.0001 ). The control group showed better performance in terms of body weight across all breeds ( P≤ 0.05). The Dolapçı and Takla breeds responded similarly to ACM and KEF treatments, having similar body weights. However, Homing breed squabs fed KEF performed better than those fed ACMs. Body weight was determined to be similar between the ACM and KEF groups in Takla pigeons.

The daily feed intake of squabs according to breed and feeding treatment is shown in Table 6.

**Table 6:** LS means and standard errors (SEs) of daily feed intake (g per squabs) of squabs according to breed and treatments.

Breed	Group	$\bar{x}$	SE
Dolapçı	CON	22.43^f^	1.18
	ACM	76.74^b^	1.02
	KEF	70.95^c^	0.97
Takla	CON	23.64^f^	1.18
	ACM	68.85^c^	1.21
	KEF	62.92^d^	1.30
Homing pigeon	CON	43.76^e^	1.30
	ACM	65.72^cd^	1.30
	KEF	82.45^a^	1.32
P	0.0144		

The columns with different letters show a significant difference between breed x treatment subgroups ( P≤ 0.05).

Feed intake varied significantly by breed, group, observation day, and their interactions ( P≤ 0.05), except for the interaction between breed and observation day ( P>0.05 ). The control group had significantly lower feed intake than the other groups ( P≤ 0.05). However, no significant difference in feed intake was observed between the ACM and KEF groups ( P>0.05 ). There was a notable difference in the daily feed intake of the Dolapçı, Takla, and Homing pigeon breeds ( P<.0001 ). Feed intake in breeds is ranked from low to high in the form of Takla (51.80 ± 0.69 g), Dolapçı (56.71 ± 0.611 g), and Homing pigeons (63.98 ± 0.753 g). In the Takla, Homing, and Dolapçı breeds, feed intake differed significantly among the CON, ACM, and KEF groups ( P=0.0144 ). In Takla and Dolapçı pigeons, the CON group had the lowest feed intake, whereas the ACM and KEF groups showed higher values. In Homing pigeons, the KEF group had the highest feed intake, followed by the ACM and CON groups.

The results of the feed conversion ratios by days 0–10, days 10–28, and the entire experimental period (days 0–28) are summarized in Figs. 2 to 4.

**Figure 2:**
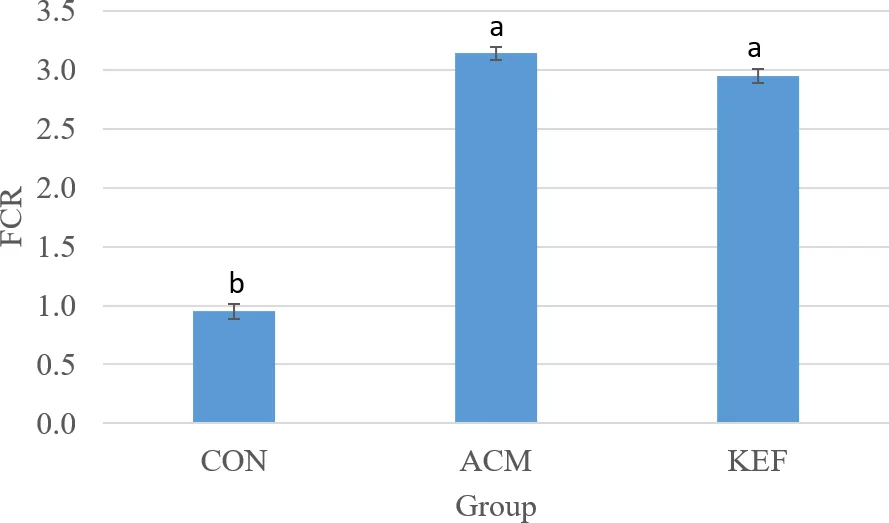
Average feed conversion ratios of the groups between the initial and 10th day of the experiment.

The FCR of the squabs differed greatly between the groups ( P<0.0001 ), but this was not influenced by their breed or the interaction between breed and group ( P>0.05 ). The CON group had the lowest FCR and therefore the best feed conversion efficiency during all three periods compared with the ACM and KEF groups ( P<0.0001 ). In contrast, the ACM and KEF groups had higher FCR values, indicating that they consumed more feed per gram of weight gain than the CON group.

During the first 10 d of the experiment, FCR was significantly different among all groups. The CON group had the lowest and therefore the best FCR (0.95 ± 0.059), followed by the KEF (2.95 ± 0.062) and ACM (3.14 ± 0.058) groups, respectively ( P≤ 0.05). Thus, both the KEF and ACM groups showed poorer feed conversion efficiency than the CON group during this period. Between days 10 and 28, the CON group again had the lowest FCR (6.10 ± 0.533), indicating the best feed conversion efficiency, whereas the ACM (13.73 ± 0.536) and KEF (13.30 ± 0.532) groups had significantly higher and similar FCR values ( P≤ 0.05).

**Figure 3:**
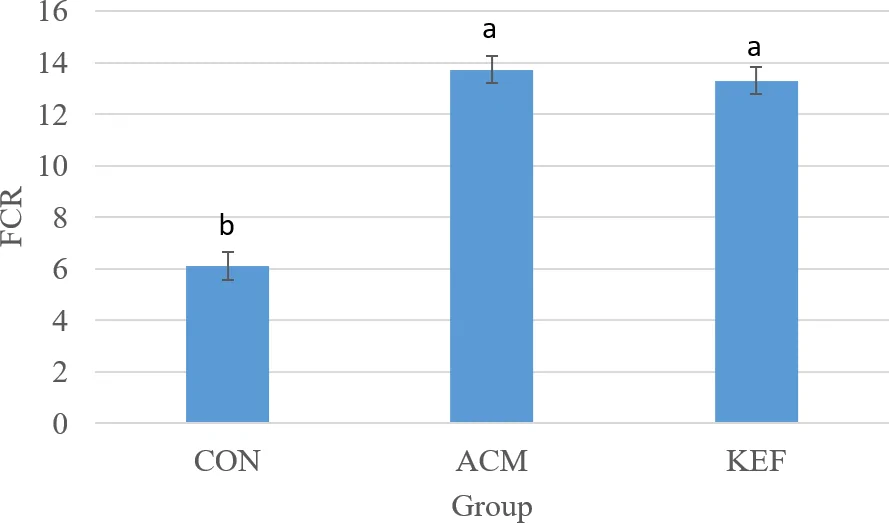
Average feed conversion ratios of the groups between the 10th and 28th days of the experiment.

During the experimental period, the CON group had the lowest overall FCR (2.61 ± 0.187), followed by the KEF (8.37 ± 0.186) and ACM (8.92 ± 0.188) groups, respectively ( P<0.0001 ).

**Figure 4:**
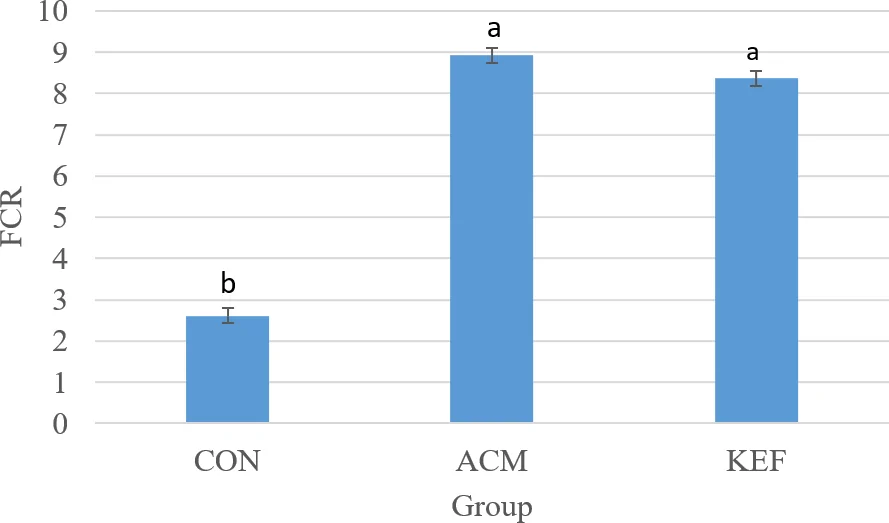
Average feed conversion ratios of the groups.

These results indicate that squabs in the CON group converted feed into body weight more efficiently than those in the ACM and KEF groups, while the KEF group showed slightly better overall feed conversion than the ACM group.

As shown in Table 7, no mortality was observed in the CON group. In contrast, mortality was recorded in both hand-fed groups, with 8 squabs (23.8 %) dying in the ACM group and 10 squabs (19.5 %) in the KEF group. However, the differences in mortality among the treatment groups were not statistically significant ( P=0.6345 ).

**Table 7:** Mortality distribution of pigeon squabs according to breed and treatments.

Groups	Dolapçı	Takla	Homing	Mortality
			pigeon	rate
CON	0	0	0	0.00 %
ACM	4	2	4	23.80 %
KEF	5	1	2	19.51 %

### 3.3 Serum biochemical parameters

The ACM group had the lowest serum ALB concentration ( P=0.0257 ). Nutritional treatments had a significant impact on serum total protein and albumin levels ( P≤ 0.05). The breed had a substantial impact on the parameters of GLU, TP, ALB, CHO, and ALT ( P≤ 0.05). The serum parameters were not affected by the interaction between nutritional treatment and breeds ( P>0.05 ). The highest concentration of serum TP was observed in the control group (Table 8).

**Table 8:** Changes in serum parameters according to nutritional treatment groups.

Group	CON		ACM		KEF		P
Item	$\bar{x}$	SE	$\bar{x}$	SE	$\bar{x}$	SE	
GLU	286.85	7.308	273.71	6.831	280.98	7.678	0.4225
TP	2.37^a^	0.056	1.97^b^	0.053	2.02^b^	0.059	<0.00001
ALB	1.50^a^	0.045	1.34^b^	0.043	1.38^a^	0.048	0.0257
CHO	281.38	9.620	257.52	9.130	273.52	10.260	0.1898
AST	139.15	6.210	130.97	5.880	127.45	6.390	0.4024
ALT	160.26	8.141	141.27	7.717	133.27	8.172	0.0606

GLU: glucose; TP: total protein; ALB: albumin; CHOL: cholesterol; AST: aspartate aminotransferase; ALT: alanine aminotransferase. The rows with different letters show significant difference between treatments ( P≤ 0.05).

The changes in biochemical parameters of serum according to pigeon breeds are presented in Table 9.

**Table 9:** Serum biochemical parameters according to pigeon breed.

Breed	Dolapçı		Takla		Homing pigeon		P
Item	$\bar{x}$	SE	$\bar{x}$	SE	$\bar{x}$	SE	
GLU	271.12^a^	6.50	272.18^a^	7.27	198.26^b^	7.99	0.0203
TP	2.36^a^	0.05	2.03^b^	0.06	1.97^b^	0.06	<0.00001
ALB	1.34^b^	0.04	1.35^b^	0.05	1.53^a^	0.05	0.0089
CHO	282.26^a^	8.52	244.11^b^	9.72	286.05^a^	10.68	0.0047
AST	132.67	5.04	130.6	5.99	134.3	7.25	0.9224
ALT	158.08^a^	6.61	130.86^b^	7.86	145.86^ab^	9.34	0.0343

GLU: glucose, mg dL^−1^; TP: total protein, mg dL^−1^; ALB: albumin, mg dL^−1^; CHO: cholesterol, mg dL^−1^; AST: aspartate aminotransferase, U L^−1^; ALT: alanine aminotransferase, U L^−1^. The rows with different letters show significant difference between breeds ( P≤ 0.05).

The lowest mean value in terms of serum GLU concentration was found to be that of the Homing pigeons, while the Dolapçı and Takla breeds had higher and similar means compared to the Homing pigeons. While Dolapçı pigeons had statistically higher serum TP concentrations ( P≤ 0.05), Homing pigeons and Takla pigeons had similar concentrations ( P>0.05 ). Serum albumin concentration was found to be lower and similar in the Dolapçı and Takla breeds when compared to the Homing breed (Table 9). The serum cholesterol concentrations of the Dolapçı and Homing pigeons were similar and significantly higher than those of Takla pigeons. The serum ALB concentrations of the Dolapçı breed pigeons were significantly higher ( P≤ 0.05) than those of the Takla and Homing breeds.

## 4 Discussion

### 4.1 Crop milk quality

As stated by Shetty et al. (1992) and Sales and Lanssens (2003), crop milk is found to contain 9 %–18.8 % CP and 4.5 %–12.7 % EE on a wet basis. In this study, it was determined that the CP content of crop milk on a wet basis according to pigeon breeds varies between 7.18 % and 8.33 %, and EE varied between 5.14 % and 5.71 %. These values fall within the range previously reported (Shetty et al., 1992; Sales and Lanssens, 2003). Crop milk is reported to contain 64 % CP and 30 % EE on a dry matter basis (Hu et al., 2016). The EE value in this study was in accordance with the value reported by Hu et al. (2016), but the CP value was lower (42.75 %). The chemical composition of crop milk is reported to vary according to nutritional treatments (Zhang et al., 2016; Chen et al., 2020a) and different incubation periods (Chen et al., 2020b). In the present study, the parents were provided with a standard diet, and there were no differences in the chemical composition of crop milk according to breed ( P>0.05 ).

The milk from the crop contains a significant amount of microorganisms, which is crucial for the squab's microbiota and immune system development, and thanks to these microorganisms, microbial fermentation occurs in the crop (Jin et al., 2020). It has been determined that the intestinal microflora of chickens fed with crop milk has developed significantly (Gillespie et al., 2012). Reports indicate that crop milk contains *Lactobacillus* spp., *Enterococcus* spp., and *Bifidobacterium* spp. It has been reported that the initial week “following” hatching is critical for the development of the gut microbiota, with the colonization of *Lactobacillus* spp. and *Escherichia* spp. during this period being important for immune development (Xu et al., 2022). Survival rates and production performance were enhanced by adding probiotics to artificial dairy products (Ding et al., 2020).

The levels of total aerobic bacteria (TAB) and yeast in crop milk did not differ significantly among the breeds ( P>0.05 ). Although no significant breed effect was detected, the relatively high within-breed variation in yeast counts may have contributed to this result. However, among the breeds, the highest LAB concentration was found with 3.96 ${\mathrm{log}}_{10}$ cfu g^−1^ in the Homing pigeons ( P≤ 0.05). The relatively high LAB abundance in pigeon crop milk, particularly *Lactobacillus* spp., has previously been reported and may contribute to the acidic characteristics of crop milk (Ding et al., 2020; Wang et al., 2023). Lactic acid bacteria use sugars in the environment to obtain nutrients and produce a significant amount of lactic acid. It can be stated that lactic acid production is also higher because this breed has significantly higher levels of LAB than the other two breeds.

### 4.2 Performance of squabs

Insufficient growth in squabs is often caused by the treatment of artificial crop milk during early growth stages (Xu et al., 2022). Those fed artificial crop milk between the 7th and 25th day after hatching had a lower body weight than those fed by their parents, as revealed by Wen et al. (2022). The study conducted by Liu et al. (2025) included squabs that were fed artificial crop milk that differed in protein levels (14 %, 15 %, 16 %) from 20 d of age onwards. According to Liu et al. (2025), the group fed artificial crop milk containing 16 % protein had a higher body weight at 28 d of age than the parent-fed (control) group. In addition to pigeons, Sklan and Noy (2005) reported that the growth performance of chickens can be enhanced by ensuring a high level of CP intake. It is stated that replacement of pigeon crop milk with artificial crop milk at an early age can lead to inadequate growth performance (Liu et al., 2025). According to the study, the parents' crop milk's CP content averaged 42.92 % in DM, while the artificial crop milk's CP content averaged 26.02 % in DM. When calculated on a fresh matter basis, the CP content of the parents' crop milk was 7.86 %, whereas the CP content of the artificial crop milk diluted 1 : 1 according to the manufacturer's recommendation was 11.66 %. Since kefir was administered at 2 % of the daily feed intake, its nutritional contribution was considered negligible. Accordingly, the group consuming artificial crop milk had a higher CP intake than the parent-fed group. Nevertheless, squabs in the ACM and KEF groups exhibited lower BWG (body weight gain) and poorer FCR compared with the control group. As reported in several studies, natural crop milk contains various bioactive components that can stimulate growth (Bharathi et al., 1997; Abdel-Azeem et al., 2016). Moreover, it is rich in *Lactobacillus* and *Bifidobacterium*, microorganisms with probiotic properties that may play an important role in the growth and development of squabs. These factors may help explain the inferior BWG and FCR observed in the ACM and KEF groups compared with the control group, despite their higher CP intake. In addition, the manufacturer's recommended dilution ratio for the artificial crop milk may also warrant further consideration. On the other hand, given the significant differences in feed intake between the control group and the experimental group, it is essential to highlight the methodology used to quantify feed intake, as this could potentially lead to data distortions. Feed intake was estimated by weighing the squabs twice daily: once in the morning before feeding and once at noon after feeding. While squabs in the treatment groups were fed twice per day under controlled conditions, those in the control group may have been fed more frequently by their parents, which could have resulted in an underestimation of actual feed intake in this group.

It has been suggested that the incorporation of probiotics into artificial crop milk during the rearing of pigeon squabs may improve their survival and performance (Ding et al., 2020). Consistent with this hypothesis, Cho et al. (2013) reported that kefir consumption enhanced growth performance in broiler chickens, and Ghasemi-Sadabadi et al. (2019) demonstrated that supplementation of drinking water with 4 % kefir improved BWG, feed intake, and FCR in broilers. However, in the present study, supplementing artificial crop milk with kefir at 2 % of daily intake did not affect squab body weight, feed intake, and FCR. Supporting these findings, Liu et al. (2025) reported that the introduction of artificial crop milk feeding from 4 d of age may result in insufficient growth, with artificial crop milk-fed groups performing worse than the control group. Similarly, Mohamed et al. (2025) observed reduced body weight gain and poorer feed utilization in squabs weaned at 0 and 7 d of age, accompanied by increased mortality rates. In this context, De-Cock et al. (1991) reported that parents produce pure crop milk only until the third day after hatching, after which its production gradually declines. Conversely, Abdel-Azeem et al. (2016) demonstrated that squabs older than 3 d of age can achieve satisfactory growth when fed artificial crop milk. Therefore, the lower growth performance observed with artificial feeding in the present study may not be attributable to a positive or negative effect of kefir supplementation per se, but rather to differences in the chemical composition and biological properties of natural crop milk, as well as the frequency and type of parental feeding. Nevertheless, a breed-dependent response to the dietary treatments was observed. In particular, Homing squabs fed the KEF diet had higher feed intake and greater body weight gain than those fed the ACM diet, suggesting that the response to kefir supplementation may vary among breeds.

High mortality rates in the ACM and KEF groups were observed. Although the rate was comparatively lower in the squabs of the kefir-consuming group (23.8 % vs. 19.51 %), it is noteworthy that the mortality rates in the treatment groups were relatively high. In their study, Mohamed et al. (2025) determined the mortality rates of squabs separated from their parents at hatching, at 7 d of age, and at 28 d of age. The respective rates determined were found to be 26.70 %, 3.28 %, and 3.80 %. In contrast, Abdel-Azeem et al. (2016) reported that the mortality rate of the squabs fed artificial crop milk formulated by the researchers was approximately 2 %.

### 4.3 Serum biochemical parameters

Glucose is a primary energy source in vertebrates (Braun and Sweazea, 2008; Sweazea, 2022). On the other hand, it has been demonstrated that birds primarily utilize fatty acids and proteins, rather than glucose, as energy sources during long-distance flights (Jenni-Eiermann et al., 2002). Blood glucose levels in birds vary according to body size, with smaller species generally exhibiting higher concentrations (Witteween et al., 2014). This finding may be related to the lower serum glucose concentrations observed in Homing pigeons, which could be attributed to their long-distance flight capacity as a breed characteristic. Conversely, supporting the observations of Witteween et al. (2014), it can be suggested that the Takla and Dolapçı breeds exhibit higher serum glucose levels due to their comparatively smaller body size relative to Homing pigeons. In this study, it is suggested that the higher serum cholesterol concentration observed in Homing pigeon and Dolapçı breeds, which are larger than the Takla breed, may be attributable to the augmented energy requirements of these breeds.

The higher serum TP and ALB concentrations observed in the CON group may be suggestive of increased anabolic activity relative to catabolic activity within the body. This finding is further substantiated by the augmented weight gain and faster growth observed in the CON group in comparison to the squabs that were fed artificial crop milk. In the study, it is seen that the values obtained from the serum biochemical parameters measured in squabs are in line with the literature reports (Orakpoghenor et al., 2021; Zhang et al., 2022). 

## 5 Conclusions

Consequently, the body weight gains and growth performance of squabs that were parent-fed for the first 3 d after hatching and subsequently hand-reared with artificial crop milk and kefir were lower than those of squabs fed entirely by their parents. According to this study, the microbial composition and bioactive components of kefir had no significant effect on the growth performance or survival rates of hand-fed pigeon squabs. Furthermore, this research has demonstrated that the intake of artificial crop milk is associated with a decline in the growth rate of squabs. While the chemical composition of crop milk remained consistent across pigeon breeds, Homing pigeons exhibited higher levels of lactic acid bacteria populations. Serum parameters, except for AST, differed according to breed, while TP and ALB were affected by feeding practices.

A more detailed investigation of the subsequent “compensatory processes” in squabs, as well as an evaluation of the effects of artificial crop milk formulations with varying nutrient compositions on growth and reproductive traits, remains a topic of interest.

## Data Availability

The data of the study are available on request from the corresponding author.
